# Mathematical foundations of life, mind and all emergent phenomena

**DOI:** 10.3389/fnsys.2026.1803434

**Published:** 2026-07-16

**Authors:** Paul John Werbos

**Affiliations:** 1 National Science Foundation, Alexandria, VA, United States; 2 Policy and Planning Committees Q2 of Millennium Project, Institute of Electrical and Electronics Engineers-USA, Washington, DC, United States; 3 Center for Large-Scale Complex Systems & Integrated Optimization Networks, The University of Memphis, Memphis, Grenoble, TN, United States

**Keywords:** cosmos, dynamical system, learning, life, machine, mind, ehtropy, reality

## Abstract

This paper gives a roadmap for filling in a new integrated worldview, from physics to emergent phenomena, presented at Werbos (2010). That worldview starts from the assumption that the cosmos we live in is a dynamical system, which obeys what I call Hard Core Einsteinian realism (HCER), which can be approximated very well in our level of life by quantum electrodynamics as formulated by Everett, Wheeler and Deutsch (EWD) (Deutsch, 1997). In principle all of the patterns which emerge in such a cosmos are fully, precisely specified by knowing the equilibrium probability distribution (equivalent to the entropy function) for that dynamical system. These functions are known precisely for a wide class of EWD and HCER theories. Thus the understanding of life, mind and other emergent phenomena in our cosmos is equivalent to knowing the parameters of the underlying dynamical law (e.g., H, for the case of EWD), and knowing useful ways to approximate what that probability function looks like. As an example, present theories of Darwinian evolution are basically just crude approximation systems for specific types pof state or pattern or object which we usually call “life.”

## 1 The opportunity: summary and objectives

### 1.1 Broader benefits and assumptions in pursuing this opportunity

The purpose of this paper is to provide an initial sketch of a very large, unmet opportunity in the field of mathematics.

As I write this paper, many nations of the world are becoming more conscious again of the great need to respect and develop the basic principles of truth and science which were fundamental to human and economic progress in the twentieth century.

There was a dramatic day when President Trump, on television, in front of Congress, tried to propose a strategy for the COVID virus based on the principles of public relations and marketability, which have become more pervasive recently on all sides around the world. It was so exciting when Congress people rebelled, when they saw directly that their own lives might be at stake, and that there are times when the basic principles of seeking the truth need to be drawn on and fulfilled, systematically and effectively. This is true in many sectors, to help us address many great challenges bigger and more permanent than COVID. It is great to see more people interested in rebuilding, revivifying and strengthening the foundations of institutions to support these goals, and integrate these pursuits across the world. But in addition to truth and science in general, we also have huge unmet opportunities to return to certain grand goals in mathematics, fundamental to the other sciences and to challenges in policy and technology. One of those great opportunities is the opportunity to develop a more integrated, unifying approach to understanding the emergent properties of certain fundamental classes of dynamical system.

To guide ourselves away from the cultural entropy we now observe at this time in the Twenty-First Century (“The New Roaring Twenties”), I propose that we remember and rededicate ourselves to certain key principles of John Von Neumann and of Hilbert, who are often viewed as the two greatest mathematicians of the twentieth century.

In his core mathematical work, Von Neumann pushed us to develop the integrative, unifying mathematical concepts necessary especially to finding real answers to the three great questions of fundamental basic science:

1. What is mind? (How can we build and understand intelligent systems, a great connected spectrum of designs or systems ranging from simple Turing machines to the most powerful universal learning machines, building on the deep, close discussions of Von Neumann, Wiener and McCulloch).
2. What is life? (For a wide class of dynamic system models of how the universe or a segment of it may work, how can we understand what types of pattern or self-organization can emerge, showing all of some of the properties we call life”?)
3. What are the underlying mathematical laws (the “law of everything” or dynamics) which govern this universe, or for the larger multiverse of which it is part? (Many still cite Von Neumann’s great Grundlagen of Quantum Mechanics, and his follow-up work and discussions with Einstein and Wigner). But massive unmet opportunities to extend his mathematical approach have begun to appear in new approaches to Quantum Information Science and Technology (QuIST), building on but going beyond the Universal Quantum Turing machine concepts of David Deutsch of Oxford.

The mathematics of dynamical systems and of approximation theory cut across these three areas, providing even more fundamental unification, and providing a way of being more explicit about connections which Von Neumann himself became ever more aware of as he learned more in life. There has been huge progress in many specialized areas addressing these three questions (along with confusions and distractions in others, as we can see in watching the COVID debates). At the present time, human understanding in science and beyond seems to be fragmenting, becoming less unified and coherent and even less sane, but the authentic and well-grounded streams of progress related to Von Neumann’s three questions provide the nutrients we need to grow a more unified and coherent mathematical understanding than was possible before this new millennium.

I hope that this paper will be just an early sketch draft of a larger, and more coherent paper with multiple authors which does justice to the new opportunity, and to the most famous contribution of the other great mathematician of the twentieth century, Hilbert. Hilbert and Von Neumann both made huge contributions to the field of mathematical operator theory, which -- like calculus itself and linear algebra, its living parents, and like the mathematics of optimization and probability theory -- are the basic tools needed to address the core of this new unifying opportunity. But Hilbert is also famous for clearly articulating a series of well-defined problems or challenges, necessary for fertilizing solid progress in many aspects of mathematics, important not only to pure mathematics but to many areas of application. The next big challenge or milestone for us all, here and how, is to write a more complete paper which more clearly enumerates and specifies the basic types of dynamical system and approximation scheme, as well as sketch theorems or conjectures which need to be proven or restated. There is a need for discussing these systems and approximation methods in more uniform notation, with clearer comparison and identification of the gaps we need to fill to provide a more unified picture.

## 2 Approximation theorems and the connection between Von Neumann’s questions

To appreciate this opportunity and what it calls for, I need to explain a bit more why I attach such importance to the word “approximation,” which some view as a lower-level dirty practical distraction, much as some people assume about the practical fields of quantum optics and empirical science in general.

When I funded work related to all three of these basic questions at NSF, I often found myself saying “All life is approximation.”

### 2.1 Question 3 law of everything

Question 3 (“the law of everything”) is not directly about approximation, of course (Deutsch, 1985). In a way, it is the exact opposite. It is our most serious effort to write down dynamical laws which hold universally and exactly, underlying everything else we see in life. At this time, the most credible, mainstream “theory of everything” (prior to unification with gravity) is the theory from Hugh Everett, Wheeler and David Deutsch that the state of our universe at any time t is defined by the value of a “wave function” psi(*x*, t), where psi is a complex valued function of L2 norm of 1, and where *x* is a point in “Fock space” [as discussed for example, in the classic text on quantum field theory by Dyson (1960)]. This current theory states that everything we see in this cosmos is the emergent outcome of the dynamical law (d psi/dt) = iH psi, where psi is represented as a vector in Fock-Hilbert space and H is a special important operator over that space. According to that theory, the primary mission of fundamental physics today is to find the exact, correct (algebraic) specification of this operator H, the “Hamiltonian of the universe.”

This paper will not get into the details of why I prefer this version of quantum field theory (QFT) over those which most people know better, because that is not really necessary here. It is a distraction from the goals of this paper. For this paper, I do propose that we give equal attention to the “Einsteinian” alternative, the hypothesis that we actually live in a Minkowski space (flat or curved) governed by Lagrange–Euler equations. More precisely, I propose that the new paper focus on dynamical systems either of the Everett/Wheeler/Deutsch variety or the Einstein variety, or more classical ODE or PDE, and some of their discrete time and/or space equivalents. The purpose of the new paper would not be to argue which of these dynamical systems actually is the truth of this cosmos, or even to explore some of the alternatives I would want to address in a paper on physics (It would be a distraction in this paper to discuss how to clean up certain ideas of Streater and Wightmann, or how to explore new directions discussed in more general terms by David Deutsch or David Bohm or Smolin or Laughlin, etc. Important as those ideas are, there is mathematics which needs to be cleaned up and organized before those alternative are more fully understood).

My current, new answer to this question, is that the universe is governed by Lagrange–Euler equations, modified by general relativity, as described in the book Classical Fields by Moshe Carmeli, modified by adding “gauge fields” to the concept of Lagrange–Euler dynamics (Carmeli, 2001).

The usual treatment of Lagrange–Euler dynamcs assumes that the universe minimizes or maximizes (or minmaxes) the Lagrangian field over all space–time. But a more complete assumption is that the universe does so subject to equality constraints, which is what the gauge fields even in Maxwell’s Laws represent. For mainstream quantum field theory, this is explained concretely in the seminal text https://www.damtp.cam.ac.uk/user/tong/qft.html. Strong new empirical data radically changes our understanding, with an increidble volume of evidence (Werbos 2026a, b).

### 2.2 Question 2—what is life?

These prerequisites are actually quite important to allow us to address question 2, “what is life?”, in a more principled and fundamental mathematical way.

After all, how can we analyze what patterns or organization can emerge in a “universe”, in a well-defined (large) dynamical system, without being explicit about what the dynamical system might actually be?

For the case of the Everett/Wheeler/Deutsch dynamical system (aka “the modern Schrodinger equation:, which takes fewer characters to type than to name), the exact solution to question 2 is already well-known. Simply look at the equation for the “grand canonical ensemble” in authoritative sources such as Chaikin et al. (1995). By the way, when I knew Chaikin, he was the lead graduate student researcher for Julian Schwinger, who shared the Nobel Prize for the canonical version of QFT, which is based on that same modern Schrodinger equation, making the further assumption that H is the “normal form” Hamiltonian. There is no randomness in that equation, and no metaphysical zero point energy terms either; contrary to popular myth, the canonical form of QFT accurately predicted phenomena like vacuum fluctuations without any need for such barnacles.

The underlying dynamical system is not stochastic, Probability is fundamental, however, in what emerges in such a system. If the state s(t) of the multiverse at time t is simply defined as the value of the function psi at time t, then traditional notation would discuss Pr(s)d^s, the probability distribution of states over the infinite dimensional measure d^s. That still is an important object to consider here, but modern empirical physics finds it useful to work with a certain function of Pr(s), the “density operator” rho, defined as,


Rho=integral overallsof(psi(s)Psi∗(s))Pr(s)d^s

See Werbos (2014), and earlier published papers it cites, for more details. The key point is that we already know what the allowed equilibrium solutions are for rho, for the egeneral case of all Everett/Wheeler/Deutsch systems. They are simply the grand canonical ensemble, for which the equation is well-known. It is basically just a generalized Boltzmann equation, giving probabilities of the form exp.(−kE - k1C1 …), where E is the energy of the state and C1 to Cn are the values of other conserved quantities.

And so, if we ask whether patterns which we call “life” have significant probability (more than the very tiny probability we would get in a “heat death” random system) of emerging for any choice of Hamiltonian H, the exact answer is already there embedded in the grand canonical ensemble.

But this is not as real answer to our question until we learn how to dig out what we really want to know, by finding a more useful approximation to the grand canonical ensemble. The key to question 2 is the search for more useful approximations. More precisely, the new paper should discuss approximation schemes not only for Pr(s) for states of the Everett/Wheeler/Deutsch dynamical system over interesting choices of H, but also for Einsteinian and simpler systems with some hope of illustrating the general basic principles and possibilities for life.

In some ways, this would build on the book Self-Organization, edited by Pribram, from Erlbaum, which starts with chapters by Prigogine and by me, posing some of the questions and issues which need to be better understood.

In addition to Pr(s) and rho, useful approximation theory should unify our use of general approximation functions f(W,s-,alpha), where W refers to a set of parameters (discrete and continuous) to be used in a general approximation, where alpha represents a (scalar) measure of the level of effort or accuracy, and where some approximation functions may refer at times to previous information s- of some kind. Different approximation functions have been used for different dynamical systems and different applications, but common notation may make it easier to compare across domains. As an example, one might approximate the state of an “infinite ocean” in which life evolves by representing it as a countable infinity of “bodies” embedded in a homogeneous “soup. Ordinary Darwinian evolution may be represented approximately as the progression of the dynamical system toward an equilibrium probability distribution of such states. Some of the Russian work on the foundations of life or artificial life try to represent cellular evolution and what microbes could be under that kind of approximation. In fact, the old cross-cutting NSF program in Quantitative Systems Biotechnology (QSB) did include this focus for research, see National Science Foundation, Quantitative Systems Biotechnology (2004). But we also begin to understand that a proper, more general understanding of “life” in dynamical systems should make room for a more general set of possible emergent phenomena; for example, see https://www.amazon.com/gp/product/B000SEGSC2/.

Also relevant is the mathematical work of Vladimir Arnold probing the possibilities for dynamical systems “between fire heat death” and “ice” (limit points), anticipating important important later work by Per Bak and Kadanoff in that zone [I have Arnold’s more general book, in that area, but for others good starting points might be Arnold (2013)].

Better, general mathematical foundations for the issue of “what is life?” would be relevant to all the many practical examples and applications addressed under Quantitative Biology, which important enablers for health, agriculture, ecology and use of bioreactors to create useful products such as alternate liquid fuels, medication and even food.

### 2.3 Question 1—what is mind?

In world cultures today, the question “what is mind?” has many different, fascinating meanings to different people. As in section (2.1), I propose that the new paper should address the more mathematical questions which follow from one approach to the broader question, following the path of Von Neumann. As in section 2.1, it should consider several important classes of systems (emergent systems) and mathematical models.

The only minds whose existence we humans can be reasonably sure of, and willing to agree with, are brains, which emerged on earth as part of the evolution of life. Let us define a mundane brain as the intelligent system embedded in such a brain, and assume that this general intelligence is governed by neural network mathematics following the path initiated in discussions by Von Neumann, Wiener and McCulloch.

Many authors like Demasio have rightly said that minds like the human mind are “embodied’; they are more than just the intelligent system as such. They also include sensory and motor connections, and a primary system responsible for important hard wired responses like pain and pleasure and reaction to the pains and pleasures of some other creatures. In my chapter in Pribram’s edited book, Brain and Values (from Erlbaum), I talk at length about the relation between the intelligence part and the other parts of the mundane mind/brain. Important as the other aspects are, I propose that the new joint mathematical paper focus just on the intelligence part. This should not be interpreted as lack of respect for the importance of the other part, but as a way of maintaining focus. In my own thinking, I now see a new path to unifying different viewpoints, a path which can unify a very hard core Einsteinian view of the physics with the many human cultures which view the human mind as more than just the brain (https://drpauljohn.blogspot.com/2026/05/back-to-reality-and-more-realistic.html).

#### 2.3.1 Mathematics of neural networks over ordinary time-series dynamical systems

Concretely, let us define a mundane intelligent system as a system which inputs an inborn cardinal utility function (the kind of utility function U discussed in Von Neumann and Morgenstern, the Theory of Games and Economics behavior), and a kind of universal learning ability to discover strategies of action to maximize the future expected value of utility. Within the fields of neural networks and of control theory, we have already developed a huge mass of designs, mathematics and applications of such systems, within the field now called “RLADP” (Reinforcement learning and Approximate Dynamic Programming). See my overview article in Werbos (2010). Many general approximation theorems already exist, addressing three types of approximation task:

1. To approximate f(x) by a general approximator, usually a neural network, by F(x, W, C), where W is a set of continuous weights W, C is a set of discrete variables (such as presence or absence of a connection) and where W and C are parameters to be adjusted to get a good approximation. The seminal theorem by Barron (1994) had a huge impact, because it provided limits on how many weights are needed to attain approximation bounds for a large class of smooth functions *f*. In Werbos (2010), I reviewed that work, and the need to consider a larger class of functions f and more powerful approximators, in order to explain many key capabilities of mammal brains, which must address experience in a world which may be less smooth,
2. To predict or model x(t) as a function of {x(tau),y(tau),tau<t}. generally by using some form of time-lagged recurrent network (TLRN). Most famous here is the work by Siegelmann and Sontag, which basically shows that TLRNs have universal approximation abilities, a whole level beyond the earlier, more famous work on Solomonoff priors, which was limited to the case of discrete finite systems without stochastic disturbance (Turing machines). This type of task includes the challenge of proving the ability to approximate probability distributions, as in the SEDP type of design discussed in the Handbook of Intelligent Control [Chapters 10 and 13 are posted at Werbos, 2010].
3. To approximate the value functions J or lambda, or even the optimal policy of action (evaluated based on results rather than nominal accuracy) in the actual overall RLADP task (White and Sofge, 1994). As an example, a paper by Kozma, Ilin and Werbos (http://citeseerx.ist.psu.edu/viewdoc/download?doi=10.1.1.823.6073&rep=rep1&type=pdf) demonstrates the need for a larger class of approximation network to get usable approximations of the value function for a simple decisiontask similar to what organisms must cope with.

The new field of mathematics needs to fill in the obvious gaps in what is known now in this type of function approximation, considering the large, general classes of systems or functions to be approximated discussed in Werbos (2010).

It needs to consider all the important general classes of approximating networks, building up from the simple kinds of recurrent network discussed there, up to approximators which exploit Euclidean translational symmetry (as in the “convolutional networks: so important to todays simple forms of machine learning), more general symmetry and graphical representation (as in “objects networks), and integration with issues of time. This “ladder” of ever more general approximation capability is the basis for my 2014 roadmap for how to build intelligence as high as that of a mouse brain (Werbos, 2014).

See Werbos and Davis (2016) for empirical evidence that mammal brains really do fit this kind of neural network mathematics, better than they fit old behaviorist types of models which assumes to sense of urpose, utility or “telos” built into such brains. Werbos and Davis describes a modle of intelligence in the mammal brain which integrates the cognitive prediction approaches described in Werbos (2010) and the memory kind of learning structures pioeneered in Hebb et al. (n.d) and in the work of Grossberg and kohonen.

For a new joint paper, however, I propose that we should try to outline as precisely as possible at this early stage two fundamental generalizations to this entire class of theorems and approximators.

#### 2.3.2 Neural networks and approximators over time-symmetric (at least not time forwards) dynamical systems

##### 2.3.2.1 Time-symmetric approximators/brains

Of course, we do not have theorems yet! The more basic motivation here is to quantify the approximation power of a more general type of neural network hardware, discussed in Werbos and Dolmatova (2016).

Ordinary simultaneous recurrent networks (SRN) implemented in ordinary time-forwards electronics may take a lot of time to reach equilibrium, especially if they are designed to “settle down” to a solution to NP hard problems. But a new class of quantum system, exploiting new time-symmetric device models (for which empirical data exists but is not yet published), can reach equilibrium between a starting time and a readout time by exploiting quantum mechanics, without requiring an actual passage of time. This has a relation as well to the new D-wave capabilities, also relevant to solving NP hard problems (such as breaking modern codes like the polar codes used in 5G communications) but somewhat less powerful and general.

Fortunately, there are many big open opportunities here which do not require understanding of quantum theory or even of the modern Schrodinger equation. In http://citeseerx.ist.psu.edu/viewdoc/download?doi=10.1.1.24.1741&rep=rep1&type=pdf, we already discussed some of the basic differences between the usual time-forwards kinds of dynamical systems and the full time-symmetric class.

As an example, for the simple case of dynamical systems over a lattice of space–time, important results can be proven for the case of a dynamical system (target for approximation) which qualifies as a local markhov process (LMP). In an LMP, Pr(phi(x,t + delt)) = f({phi(x + delx,t)}, where delx is a minimum move in space. But in a local Markov Random Field (LMRF) over space time, Pr(phi(x,t)) is specified as a function of phi at all points in the neighborhood of (x,t), in recent past or future and present. The LMP class of systems is a proper subset of the LMPR, in the same way that feedforward neural networks containing finite time lags are a proper subset of TLRN. See Werbos (2014) and Werbos (2021) for examples of LMRF models, over continuous Minkowski space, which predict that certain kinds of components of optical computers may have properties different from what traditional LMP models predict, and predict that we can build computers with powers that classical ones (and traditional quantum computers) cannot have.

This is relevant to Von Neumann’s second question, as follows. Classical earth-based Darwinian evolution is a feedforward process (which can be described by various approximation models) which takes many, many generations to converge. But approximation theorems relevant to this more general class of systems might allow much faster convergence or “evolution” (emergence of life types of patterns) in ecologies or universes where time symmetry comes into play. It could be possible that some types of cosmos system, like what some models for dark matter and energy might allow, would allow the evolution of lifelike patterns like “solar system noospheres”, without then requirement for trillions of years of evolution. In any case, the mathematics of what might be possible, under what conditions, is worth working out.

##### 2.3.2.2 Mind-like higher patterns in grand canonical Boltzmann probability density operators

The key idea is to try to extend what we have learned from approximating the Bellman equation (and its close relatives like the Pontryagin equation) to approximating the most modern, mainstream version of thermodynamics, given in https://www.amazon.com/Principles-Condensed-Matter-Physics-Chaikin/dp/0521794501/.

It should be noted that there is an exact mapping from probability distributions of states of a PDE system to this kind of density operator (Werbos, 2026).

Many people, in staring at the usual modern “Schrodinger equation (psi dot - i H psi)”, assume that nothing brain-like could emerge from such a system. But there are interesting properties shared by tis Boltzmann operator and the Bellman operator. The story of chaos theory warns us that “simple” looking systems like ODE can generate properties far more interesting than anyone imagined, before further study. My claim here is that these more complex dynamical systems -- nonlinear Lagrange–Euler systems over Minkowski space or curved Minkowski space, and psi dot = i H psi -- sometimes generate much MORE interesting emergent behavior, interesting in way which may even mirror the kind of complexity and “reflection” or even synchronicity phenomena we see in complex intelligent systems. To better understand such Boltzmann operators, for interesting choices of H, requires improving out ability to approximate such operators, much as we use neural network mathematics to approximate and make sense of what an organism may see in its environment.

Just how far does could the phenomena of synchronicity shape the patterns of evolution, as in question (2)? To what extent could the phenomenon of gating and object mapping to fields, present even in mouse brains, be reflected even in the reflections and resonances of large scale patterns in our cosmos? The previous issue of this journal summarizes the levels of intelligence which can be built on ordinary classical computing hardware or organic brains build on classical physics.

##### 2.3.2.3 Quantum intelligence beyond the Turing machine

**Figure 1:**
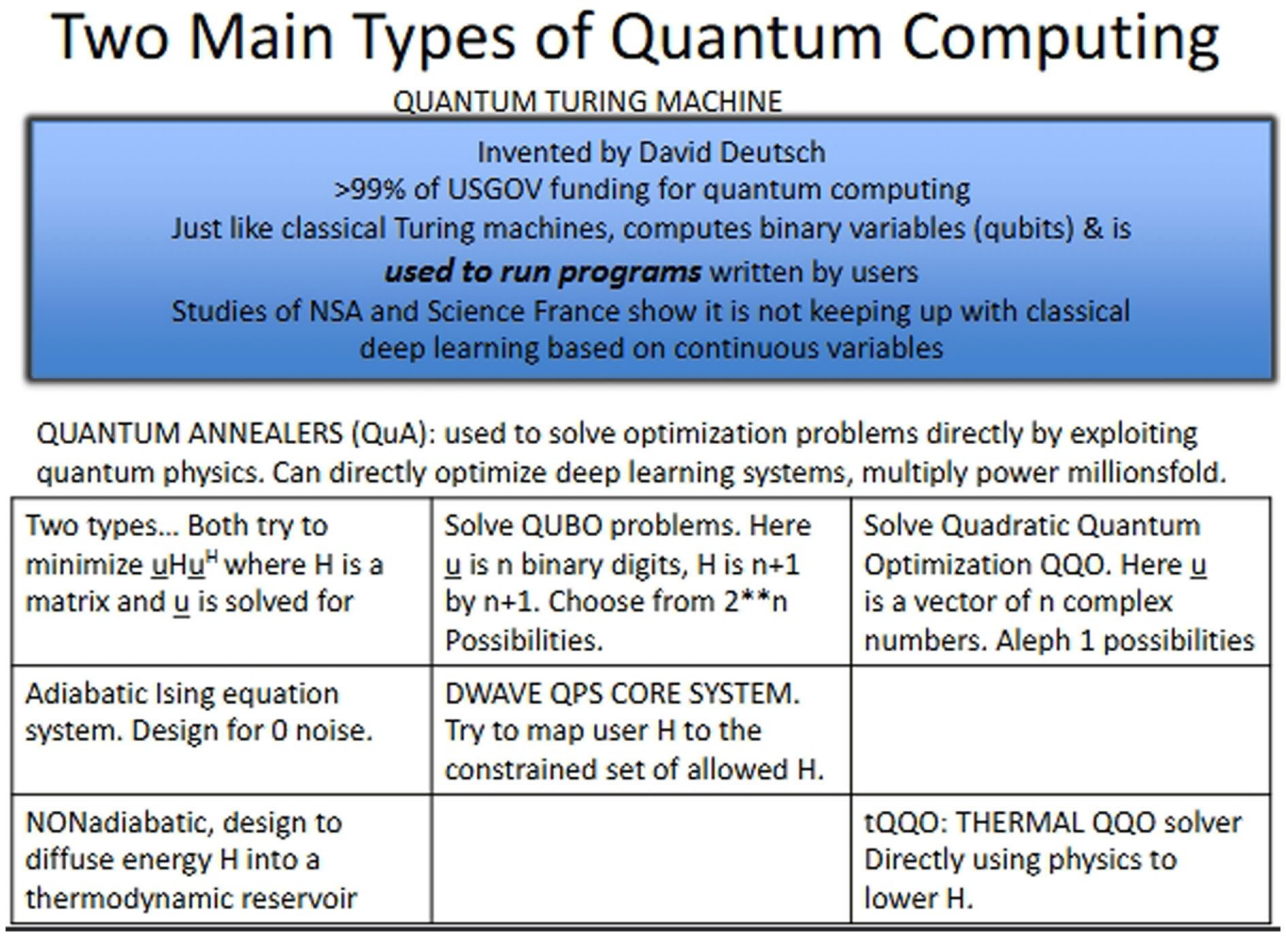
Map of types of quantum computing.

For many years, engineers working on a new kind of computing hardware, the quantum Turing machine, have demonstrated modest improvements over classical computing (Figure 1). Specialized neural network hardware has generally done better. However, in 2025 a patent was granted to a new type of quantum computing, which applies quantum superposition directly to the optimization calculations which underlie deep learning. Detailed papers are in press from USPRO, for thermal Quantum Annealing (tQuA).

## 3 Connection to network and graph theory

One of the core issues here is how to approximate the uncountable infinity of possible states of a vector field phi(x), at least for field states which obey some continuity condition.

One key approach to this approximation issue is to define classes of network or graphical models, and mappings from them to the larger continuous space. One key idea is that the set of field states which are images (mapped to) of a network state may be dense in the space of field tstaes, just as rational numbers are dense in the space of real numbers. The possible types and powers of such approximations are a major issue to be explored.

For issues like thermodynamics and life, the core issue may not be the larger uncountable infinity of possible states {phi(x)}, but the uncountable infinity of states fitting a set of Lagrange–Euler equations (though if it concerns approximation of the state at one time, this may not be such a big difference in the type of approximations).

There is a huge variety of TYPES of dynamical system, of course, on the usual spectrum from “Ice” (fixed point equilibrium types) to “fire” (heat death type), with interesting patterns, life and turbulence mainly involving possible systems between these extremes.

In chaos theory, people often begin by considering simple linear systems, and the changes in emergent behavior which result as one adds moe and more coupling. With PDE systems, and the important special case of Lagrange–Euler systems, a similar continuum is important. (Of course, Arnold and Bak and K… work on turbulence is an important foundation).

One obvious type of approximation is a countable infinity of “objects” defined as patterns (perhaps with a few arguments specifying attributes and location) located at a countable set of points.

A key point here is to find a more formal connection to object nets and the kinds of approximations we know that the brain uses. Cam thermodynamics be made at least as smart (as good at approximation) as a basic mammal brain? Translation scanning and gating is a crucial part of that, of course. APPLYING object nets to PDE output data or solutions is part of that. Tilde and caret as the two foundations of it all. And then, reemergence of Complexity: how a lattice of objects can be important in brains (as gating defined such hierarchies implicitly, but with recursion in what ways?)… and then useful provable in thermos modeling approximation.

## 4 A hint of some possible approaches

In trying to explain some of this to folks less versed in mathematics, I recently wrote:

Perhaps I will finish writing a partial draft which will really explain part of this. But for now. somehow I feel called to mention a practical little example, on the outskirts of intelligent systems, which was crucial to me in really seeing how partial gating works. It seems like a simple piece of engineering, but again and again I have been amazed at how simple pieces of GOOD engineering can manifest very important general principles.

The example comes from the field of energy system modeling. ONE of the primary (mass action) roles of cerebral cortex, after all, is to predict or model the environment of the organism as it appears on the “movie screen of the brain,” the thalamus. Prediction or modeling is a very fundamental component or aspect of any intelligent system.

From 1979 to 1989, I worked in a place charged with developing and using the main official models of the US and world energy economy in the US government, EIA/DOE. We spent lots of energy studying and advancing the art of how to build good predictive models of any system described by streams of time-series data. But we ALSO spent time on INTEGRATION, how to build a system which could COMBINE multiple models of multiple subsystems, to work together effectively (https://www.osti.gov/biblio/6288212/). Many of the techniques we studied are actually more powerful and general than any of the data analytics and policy models we ead of today. It is really sad to see models of COVID and of coming economic changes which fall far short of the quality we once had.

Back in those days, the Electric Power Research Institute (EPRI) created a vision of a new, future type of modeling system, which would be far more powerful and agile than what we had in those days, In a way, it entailed developing a whole ECOLOGY of modules.

WHY spend hours of (then expensive) computer time running the most precise, detailed model possible of X, when, for a given application, it would be good enough to use reduced form mirror of that best model? PARTIAL GATING, in essence, is the relation between the big X and the reduced form, to be connected of course with modules which integrate through multiple gates in a larger system of integrated modules.

It is ever so simple, in a way.

The mathematics of deciding whether two modules are CONGRUENT with each other, where one module can effectively approximate another, is very fundamental. For many years, people working in practical neural network engineering (back in the days when computer scientists mostly felt there is no such thing as a general learning neural network) paid special attention to the approximation theorems of Andrew Barron of Yale, showing how ANY smooth function can be approximated at bounded cost by a certain simple type of neural network, That type was not powerful enough to approximate the kinds of mappings or relations we encounter in making decisions in life, like how to navigate a cluttered space, but with Kozma and Ilin I later showed how to train a more powerful type of neural network.

I eventually provided a whole LADDER of worlds or primary functions we might try to approximate, and more complex types of neural network capable of approximating them.

And so… in an ideal world, I feel we may be ready to take that further, There are more general approximation theorems possible, which could unify this whole area, and also incorporate simultaneity ACROSS time, as in the kind of equilibrium between past and future which the Everett/Wheeler/Deutsch formulation of modern quantum field theory (QFT) assumes (Deutsch, 1985).

Some folks on this list have been raised to believe in many fundamentalist religions, such as the belief in cartesian dualism and “god in a [pair of sunglasses (the Copenhagen model of measurement from the 1920s which remains very influential in tribes which have yet to learn modern mathematics let alone mathematical physics or engineering)]. In truth, I do not really believe that the modern “Schrodinger equation (psi dot = i H psi) is the total dynamic law of entire cosmos or multiverse, but it is the best basis we have right now to make better real sense of the emergent phenomena we have around us, on all scales of human experience. To ignore what it can tell us is just neurotic avoidance behavior. (Yes, we see a lot of that kind of behavior lately in very high places, but that is no justification for imitating it).

The more general approximation theorems with partial gating would not only fit the kind of “matching which we see in the mundane brain (beautifully described in Pribrams bopk Brain and Perception) but also the capabilities of fifth generation analog quantum computing learning systems (previewed in the paper by Werbos and Dolmatova in *Quantum Information Processing*). Ordinary Darwinian evolution is basically a slow, feedforward process to reach an equilibrium thermodynamic distribution, but quantum evolution is the time-symmetric equivalent, much faster, and one way to try to understand not only the evolution of noospheres but of the patterns in the larger canonical Boltzmann distribution which is full of its own type of “matching” or “reflection.”

It is not just a poetic accident that the words “mirror” or “reflection occur over and over again in the deepest mystical literature as well. I cannot help recalling “the mirror or Ameraterasu” for which I posted a photo link at Werbos (2010).

Of course, the degree of congruence is always approximate in brains (and even in thermodynmaics at a cosmos level). Great work on neural networks and on consciousness osmetimes seems like a hall of mirrors. The less evolved system of policy intelligence we see even in the best of governments and human organizations is sometimes more like a hall of fun house mirrors, where the imperfections can be devastating. A lot of the “fake news paranoia left and right has that kind of flavor, and is truly dangerous to the survival of all of us. Perhaps we need more of a a different kind of mass action, more like Freeman’s than like the left or the right or other such dogmas negligent of the need for truth, science and yes, mathematics. I really applaud the person Robert and I know, who put a huge statue of Von Neumann in the center of her living room (and did friend me on Facebook).

## Data Availability

The original contributions presented in the study are included in the article/supplementary material, further inquiries can be directed to the corresponding author.
