# Supplementary material for: Mathematical foundations of life, mind and all emergent phenomena
Source: Front Syst Neurosci. 2026 Jul 16;20:1803434. doi: 10.3389/fnsys.2026.1803434 (PMC13422456; doi:10.3389/fnsys.2026.1803434)
Supplement: Supplementary file 1 [file Presentation_1.pdf]

Seeing the Sky: New Quantum Hardware Offers Millions-Fold More Power

* **Why we need it:** seeing the sky and human survival
* **The old AI revolution,** now just started:

  from sequential computers Turing machines) and programs to deep learning, cognitive optimization and prediction (COPN), nvidia type chips

  -- chips implement classical physics
* **Thermal quantum annealing (tQuA):** uses quantum superposition to run millions of machines in parallel in COPN applications, like "seeing the sky"

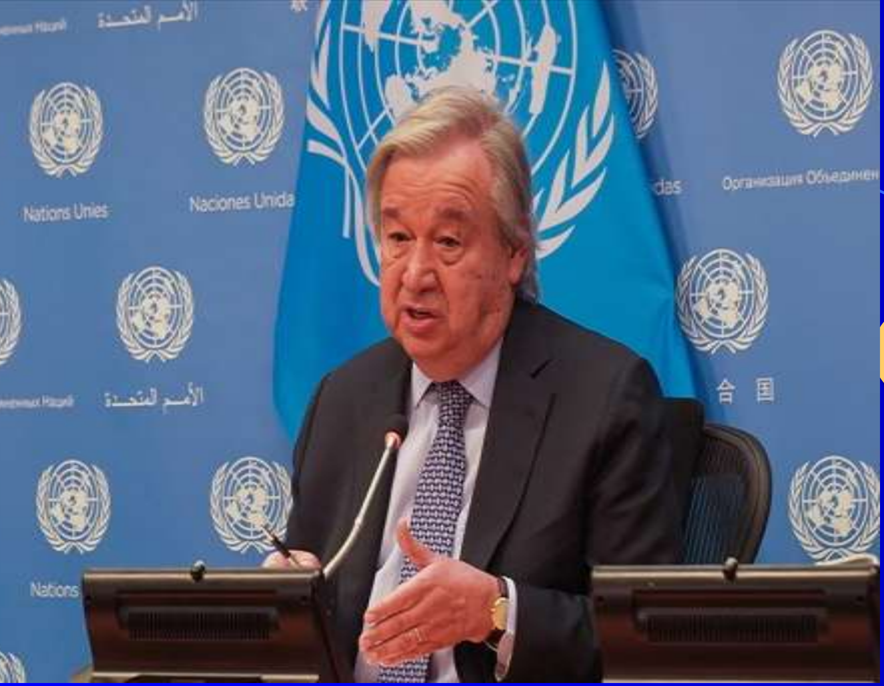

UN 7/2023 AI Security Council: Open Transparent Seeing The Sky To Prevent WW1

NDTV WORLD

Home | World News | Trump's Dream, China's Reality: Beijing Develops Planet-Wide Defence System

Trump's Dream, China's Reality: Beijing Develops Planet-Wide Defence System

According to the South China Morning Post report, scientists in China have deployed a working prototype o[illegible] their defence system. The system reportedly uses diverse sensors in space, the ocean, the air and on the ground to identify and analyse potential threats.

Edited by: Sanstuti Nath | World News | Oct 14, 2025 16:32 pm IST

7 Grand Challenges This Century

- Four Great Fears ("Existential Threats") : **SURVIVAL** **See build-a-world.org**
  - Internet/AGI/IOT if we fail to reach Sustainable Intelligent Internet (SII) **--- Depends on mind, intelligent systems**
  - Climate extinction, all of our species and many others
  - Misuse of nuclear tech, or biotech (e.g.werbos.com/NATO_terrorism.pdf and blogspot posts)
- Two Great Hopes: **GROWTH**
  - Quantum leap in attaining human potential, including our connections to each other & our noosphere (soul,qi)
  - Economically sustainable settlement of space (e.g. NSS)
- SITUATIONAL AWARENESS FOR HUMANS IN COSMOS: New tech (QAGI/axions) to see the sky

MOST URGENT

- WAR: Patriot missiles miss ten times as many missiles as the stage 2
- of the Balakrishnan design would. China and India have better. US held back by the stakeholder system. Advanced RLADP applies to drones, not just missiles. Security Council 2023 AI session still points to best hope for sustainable survival, but only if paths to advanced technology included better.
- Currency: Federal reserve UNDERSTATES need for US caution, because US cannot just print money now. True digital reserve apps (known in China, far more secure than any bit coin type system) will change the world dramatically and suddenly. Only better international collaboration can present disaster.
- Cybersecurity everywhere – machine rules to prevent corruption by human COI, quantum and rainbow partnership

New 2D Nanotech PLUS QAGI permits seeing the sky in nuclear bands, from dark matter to attacks to qi (Schwinger, Teller)

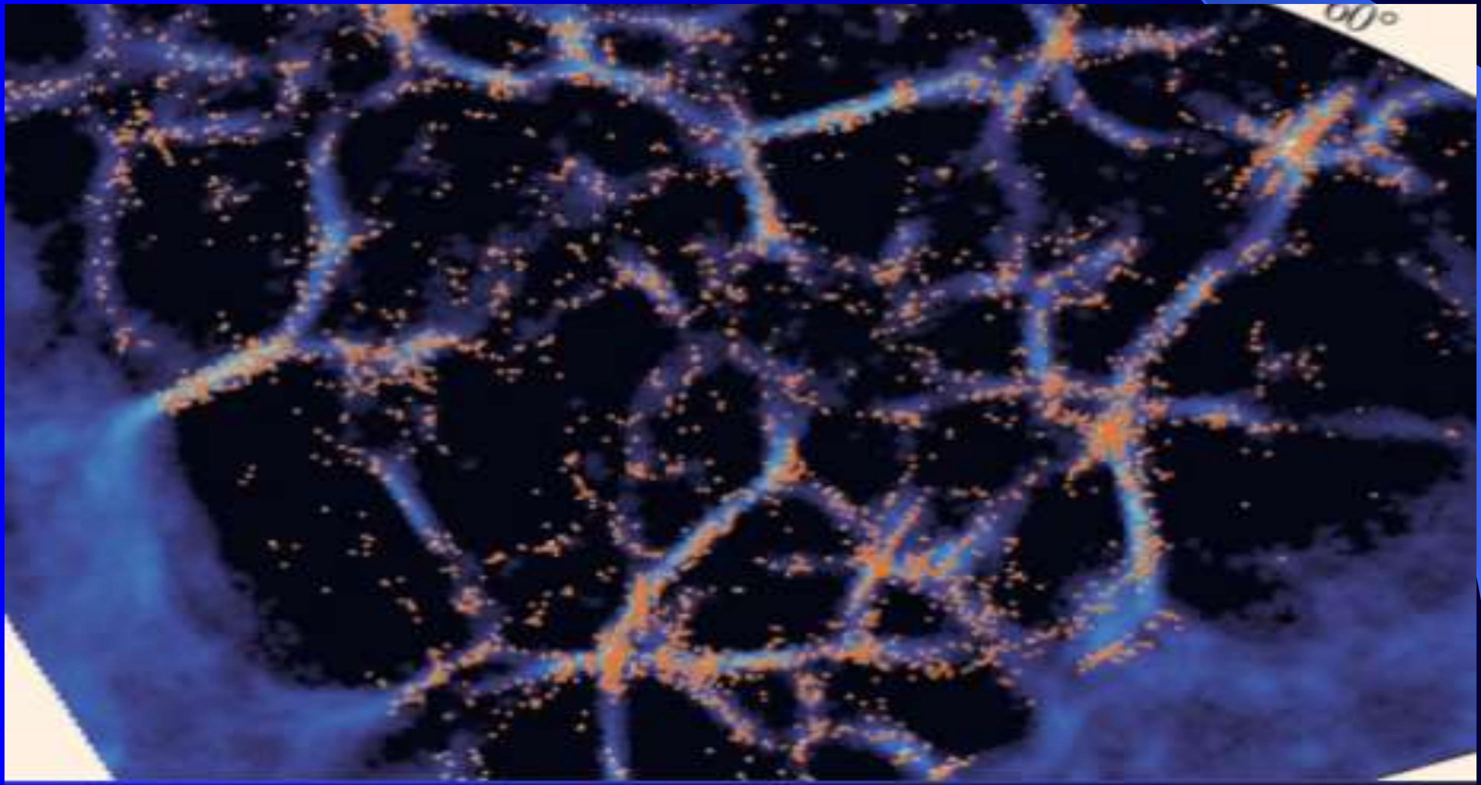

Three Levels of "AI"

- GAI – Generative AI, chatbots. Started in Eliza, MIT, 1966 – now expanded using early, simple AGI underneath IN US (China ahead). See OpenAI https://www.youtube.com/watch?v=26sGJESV65E UNCONTROLLABLE CHAOTIC started already
- Artificial General Intelligence (AGI), area I led at NSF 1988-2015, deep learning and RLADP. My IEEE Rosenblatt Award 2022. Universal function max/min, prediction, decision&control by RLADP
- Quantum AGI (QAGI), new patent, millions of times better shown already, opportunity for you

Artificial General Intelligence (AGI):

- A **Tsunami** of New Waves Coming

AGI + INTERNET OF THINGS (IOT)

- IS COMING but who will build the best and safest new AGI? What is AGI?

Is it AGI coming?

Is it about Slaughterbots? (Stu.Russell)

Is it IOT coming?

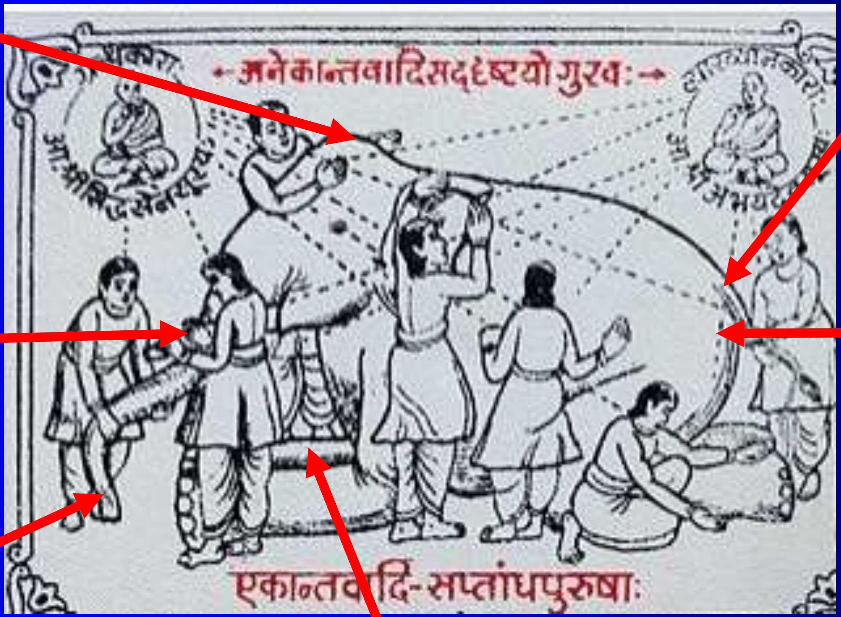

Is it what they will do with your data?

Is it what fake news and lack of truth could do to human society?

Is it what they do to your jobs & work?

Neural Net Revolutions: From BP (neural and fuzzy) to Quantum Artificial General Intelligence QAGI

- 0: Von Neumann,Wiener, McCulloch; Hebb
- 1: IEEE ICNN 87/88 (Hecht-Nielsen), NSF88 (me)
  - Backpropagation Thru Time (Proc IEEE 1990)
  - Neurocontrol: From Adaptive Control to RLADP
- 2: NSF COPN 2008: Ng/LeCun to Google Tensor Flow, “Deep Learning Revolution”, More NIPS&CompSci
- 3: WCCI2014: Beijing: Roadmap to Mammal level AGI, NSFC /CAS and Werbos/Davis (2016), patents
- 4: TODAY: QAGI, quantum RLADP (sky, cybersecurity, global power grids…)

Never forget the grand challenge for this century!

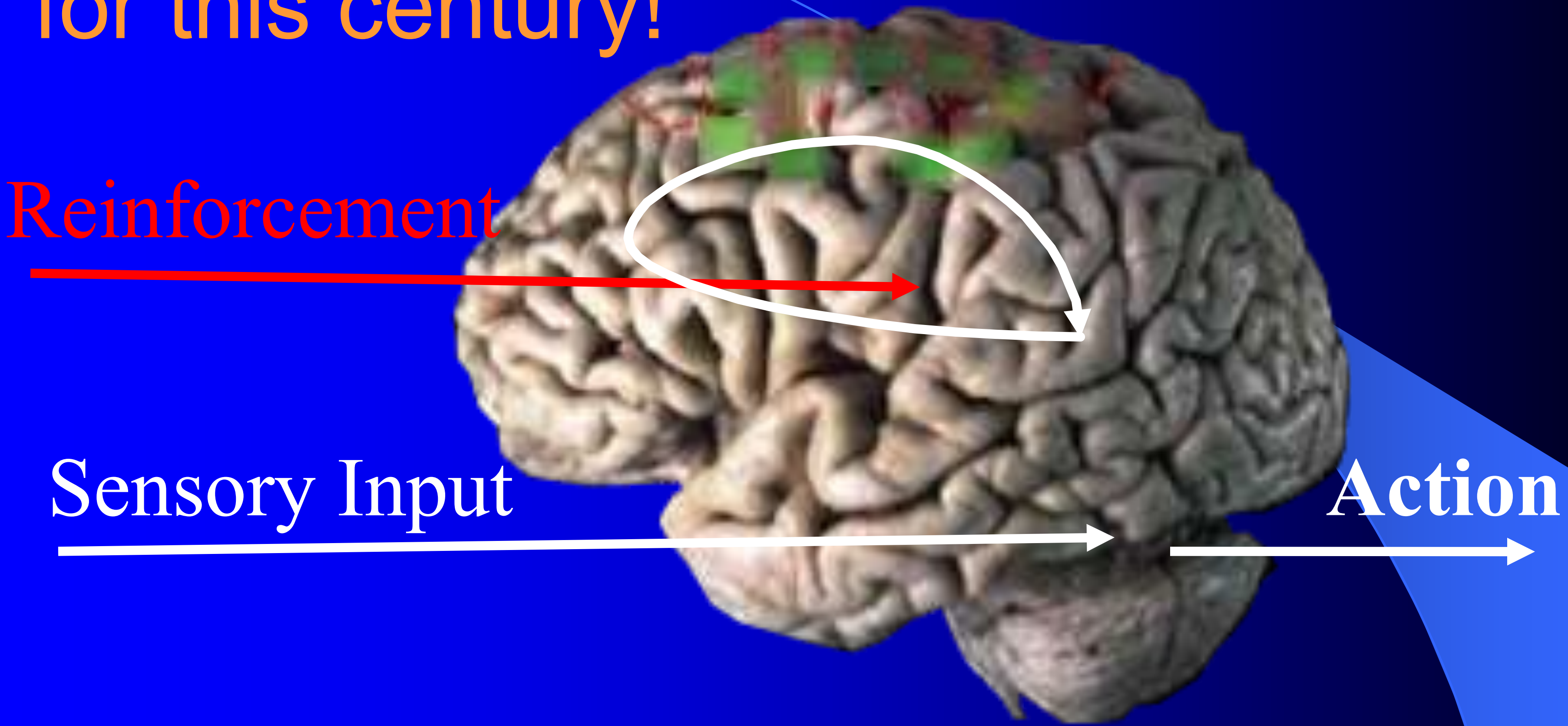

Brain As Whole System Is an Intelligent Controller

-- Mouse maximize probability of survival among other things

-- Lots of animal behavior research

-- Lots of recent motor control research (UCSD…)

True AGI makes DECISIONS Based on foresight and understanding by neural network & Values U/J (as do brains, alpha Go)

See COPN

at www.nsf.gov

Memory

Clustering

. . .

Pr(A|B)=Pr(B|A)* Pr(A)/Pr(B)

Prediction

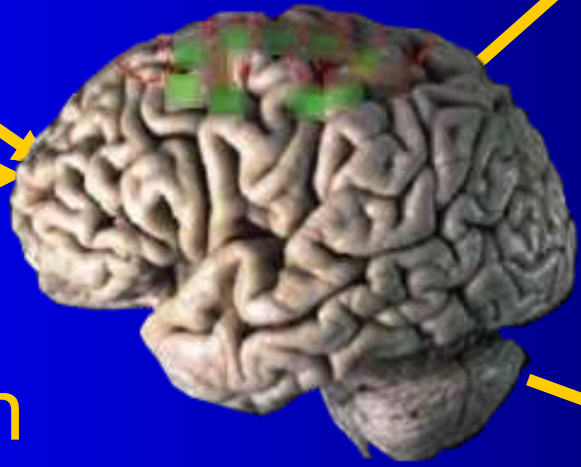

Optimal Decision

J(t)=Max<J(t+1)+U>

$$\frac{\partial^+ z_n}{\partial z_i} = \frac{\partial z_n}{\partial z_i} + \sum_{j=i+1}^{n-1} \frac{\partial^+ z_n}{\partial z_j} \frac{\partial z_j}{\partial z_i}$$

Von Neumann U = telos

Important future applications

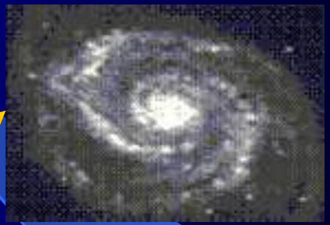

Space

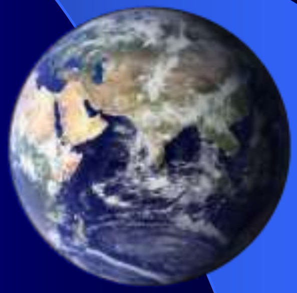

Sustainability

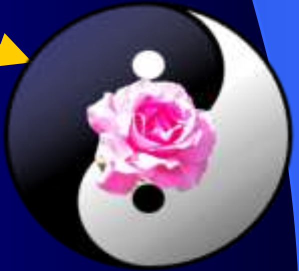

Human Potential

Revolution #2 STARTED IN 2008
www.weforum.org

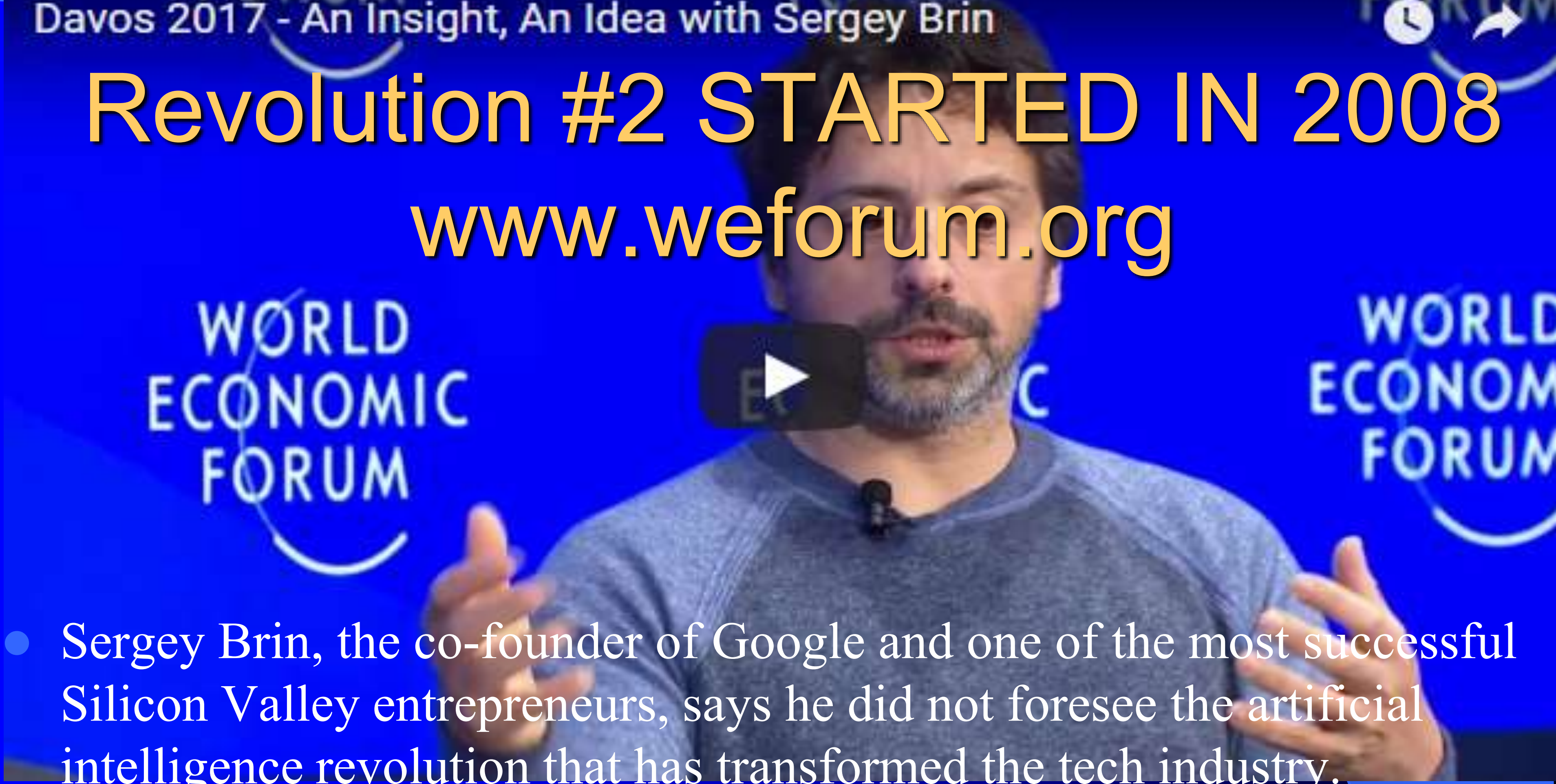

- Sergey Brin, the co-founder of Google and one of the most successful Silicon Valley entrepreneurs, says he did not foresee the artificial intelligence revolution that has transformed the tech industry.
- "I didn't pay attention to it at all, to be perfectly honest," he said in a session at the World Economic Forum's Annual Meeting in Davos. "Having been trained as a computer scientist in the 90s, everybody knew that AI didn't work. People tried it, they tried neural nets and none of it worked. (Deep Mind like D-Wave known, not enough.)

New Performance Breakthroughs in Prediction/Recognition by Ng&LeCun

Audio

TIMIT Phone classification	Accuracy
Prior art (Clarkson et al.,1999)	79.6%
Stanford Feature learning	**80.3%**

TIMIT Speaker identification	Accuracy
Prior art (Reynolds, 1995)	99.7%
Stanford Feature learning	**100.0%**

Images

CIFAR Object classification	Accuracy
Prior art (Yu and Zhang, 2010)	74.5%
Stanford Feature learning	**75.5%**

NORB Object classification	Accuracy
Prior art (Ranzato et al., 2009)	94.4%
Stanford Feature learning	**96.2%**

Video

UCF activity classification	Accuracy
Prior art (Kalser et al., 2008)	86%
Stanford Feature learning	**87%**

Hollywood2 classification	Accuracy
Prior art (Laptev, 2004)	47%
Stanford Feature learning	**52%**

Multimodal (audio/video)

AVLetters Lip reading	Accuracy
Prior art (Zhao et al., 2009)	58.9%
Stanford Feature learning	**63.1%**

Other unsupervised feature learning records:
Different phone recognition (Geoff Hinton)
PASCAL VOC object detection (Kai Yu)

Andrew Ng

New world records (under NSF COPN) using relatively simple neural networks with a symmetry addition…

#3: WCCI2014 Beijing: A Roadmap to Mammal Level AGI in Classical Computing

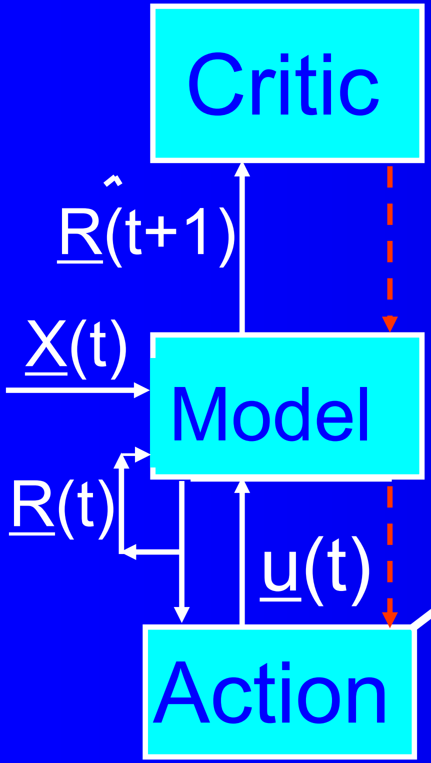

0. Vector Intelligence – HDP, DHP, GDHP, etc.

1. First ever system which learned master class chess Fogel, Proc IEEE 2004

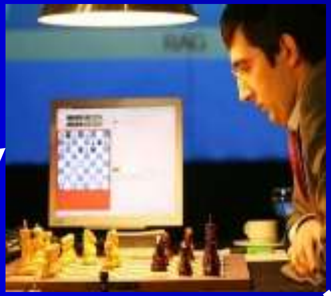

Add new spatial complexity logic (ObjectNets +…, Suitable for CNNs)

Add ability to make Decisions, plays (Modified Bellman eqs for Multiscale t.)

2. reptile

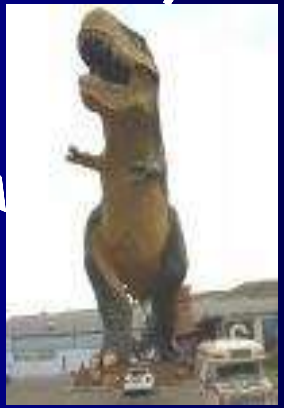

Add Creativity System (Cognitive map of space of possible decisions)

3. Mouse

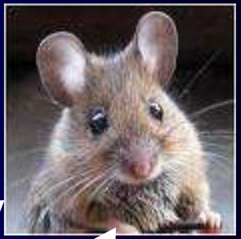

Two Main Types of Quantum Computing

QUANTUM TURING MACHINE

Invented by David Deutsch
>99% of USGOV funding for quantum computing
Just like classical Turing machines, computes binary variables (qubits) & is ***used to run programs*** written by users
Studies of NSA and Science France show it is not keeping up with classical deep learning based on continuous variables

QUANTUM ANNEALERS (QuA): used to solve optimization problems directly by exploiting quantum physics. Can directly optimize deep learning systems, multiply power millionsfold.

Two types… Both try to minimize $\underline{u}H\underline{u}^H$ where H is a matrix and $\underline{u}$ is solved for	Solve QUBO problems. Here $\underline{u}$ is n binary digits, H is n+1 by n+1. Choose from 2**n Possibilities.	Solve Quadratic Quantum Optimization QQO. Here $\underline{u}$ is a vector of n complex numbers. Aleph 1 possibilities
Adiabatic Ising equation system. Design for 0 noise.	DWAVE QPS CORE SYSTEM. Try to map user H to the constrained set of allowed H.	
NONadiabatic, design to diffuse energy H into a thermodynamic reservoir		tQQO: THERMAL QQO solver Directly using physics to lower H.

First Generation QuIST came from "Multiverse" physics: the most plausible mainstream theory of everything (Everett/Wheeler/Deutsch/MQFT)

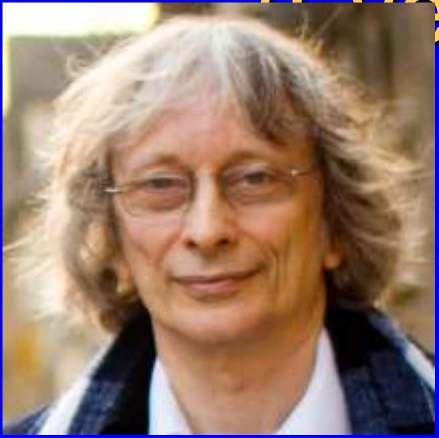

QTM:
Quantum
Turing
Machine

$$\dot{\psi} = iH\psi(X,t)$$

$$\psi = \psi_0 \oplus \psi_1 \oplus .... = \psi(FS)$$

$$\psi_1(x_1,s_1), \psi_2(x_1,x_2,s_1,s_2)$$

- We live in Fock Space (FS), an infinite dimensional space with multiple copies of all of us. WE are "macroscopic Schrodinger cats."
- Parallel computing: use thousands (Q=10) or trillions (Q=40) of copies of the SAME chip at once

2022: QUANTUM HARDWARE: From Phase 1 (QTM) to Phase 3 (tQuA) to remake the world

- Phase 1: David Deutsch, Quantum Turing Machine – well funded but limited. Just as Neural nets give huge power more than old sequential computing, we can do much better than QTM in the same way
- Phase 2: Dwave: Optimization include neural nets, AGI, BUT PHYSICS WAS weak, not so many Schrodinger Cats.
- Phase 3: With QQO demos and Hyland, you could share the IP for what runs the whole new world, QAGI. Needs sims of needle in haystack examples.

THREE LEVELS OF tQuA Thermal Quantum Annealing Technology

- 1. True Quantum Quadratic Optimization – the same task at core of Dwave system, but use of quantum thermodynamics allows orders of magnitude better capability per dollar. Enough for patents, quantum simulations.
- 2. Use this core for the nonlinear learning problems at core of best intelligent “AGI” decision systems, quantum RALDP

  Immediate applications: cybersecurity, energy systems
- 3. Optimize actual physical systems to optimize physical results, Quantum Annealing of Things QuATh
- These are also prerequisites to further possibilities, physics

Next, just backups for questions

tQuA versus DQuA: true annealing versus Bianchi

- Bianchi assumes a QuA core made up of signals y_1 to y_n, governed by Schrodinger equation $dy_i/dt = H_{0i}\, y_i + \alpha \text{ sum } (H_{0ij} y_i y_j)$
- "DQuA" Bianchi annealing just adjusts α
- True physics annealing would keep $\alpha=1$, but use COUPLING to outside (heat) reservoir, uses thermodynamics to SHED energy, minimize H, and suitable hardware
- Hardware should be low noise but is not as sensitive to decoherence as QTM because of no need to preserve initial conditions

tQuA: CAN we do it? Three stages of RD&D called for, for high Q to min or max, THREE tasks:

- Endogenous scoring: assume user goal is encoded into H. A quadratic optimization system, like the core of Dwave but true Q. Dwave interfaces and iteration strategies still can apply. Must prove basic thermodynamics for available reservoirs.
- Internal Coupled Scoring. A process inside the big computer box can input u, output a score which may be a complex function of u. Same task goal as Dwave, etc.
- Quantum Annealing of Things (QuATh): Instead of doing quantum superposition on a process inside the box, do it on a physical plant.

NSF88 Workshop: Miller, Sutton and Werbos MIT Press

NEUROCONTROL: neuroadaptive control to RLADP

RLADP: Reinforcement learning to adaptive critics to adaptive dynamic programming

First Model-based RLADP '87 led to the workshop…

IEEE TRANSACTIONS ON SYSTEMS, MAN, AND CYBERNETICS, VOL. SMC-17, NO. 1, JANUARY/FEBRUARY 1987

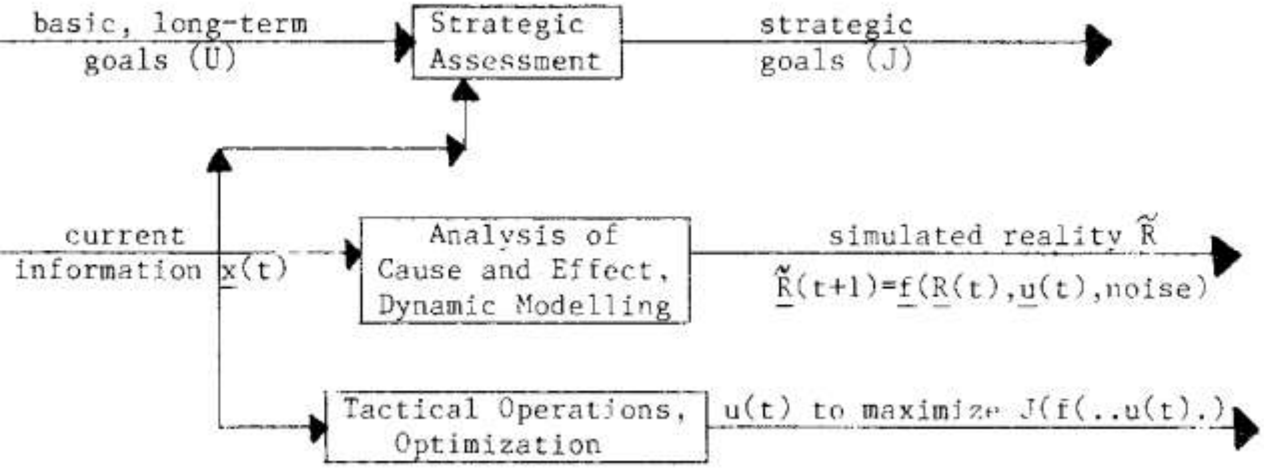

Fig. 1. Three core components of any intelligent system (J, f, u).

Goggle Tensor Board: Use of Gradients at the Core of Deep Learning

TensorFlow ™ GET

Version: r0.12

Variables: Creation, Initialization, Saving, and Loading

TensorFlow Mechanics 101

Code: tensorflow/examples/tutorials/mnist/

Training

The `training()` function adds the operations needed to minimize the loss via Gradient Descent.

Firstly, it takes the loss tensor from the `loss()` function and hands it to a `tf.scalar_summary`, an op for generating summary values into the events file when used with a `SummaryWriter` (see below). In this case, it will emit the snapshot value of the loss every time the summaries are written out.

```
tf.scalar_summary(loss.op.name, loss)
```

Next, we instantiate a `tf.train.GradientDescentOptimizer` responsible for applying gradients with the requested learning rate.

```
optimizer = tf.train.GradientDescentOptimizer(learning_rate)
```

Adaptive Dynamic Programming (RLADP) maximizes utility over time funded by NSF Engineering 1988-2021

Model of reality

Utility function U

Dynamic programming

$$J(\mathbf{x}(t)) = \underset{\mathbf{u}(t)}{\mathbf{Max}} \langle U(\mathbf{x}(t),\mathbf{u}(t)) + J(\mathbf{x}(t+1)) \rangle / (1+r)$$

Secondary, or strategic utility function J

Bellman equation: The stochastic case as needed to maximize the probability of survival

Grid is more complex than Alpha Go: Requires Lewis and Liu, RLADP Handbook, IEEE

"NSF is currently supporting research to develop a '4th generation intelligent grid' that would use intelligent system-wide optimization to allow up to 80% of electricity to come from renewable sources and 80% of cars to be pluggable electric vehicles (PEV) without compromising reliability , and at minimum cost to the Nation (Werbos 2011)."

obamawhitehouse.archives.gov/sites/default/files/

microsites/ostp/nstc-smart-grid-june2011.pdf

(or search on White House smart grid 2011)

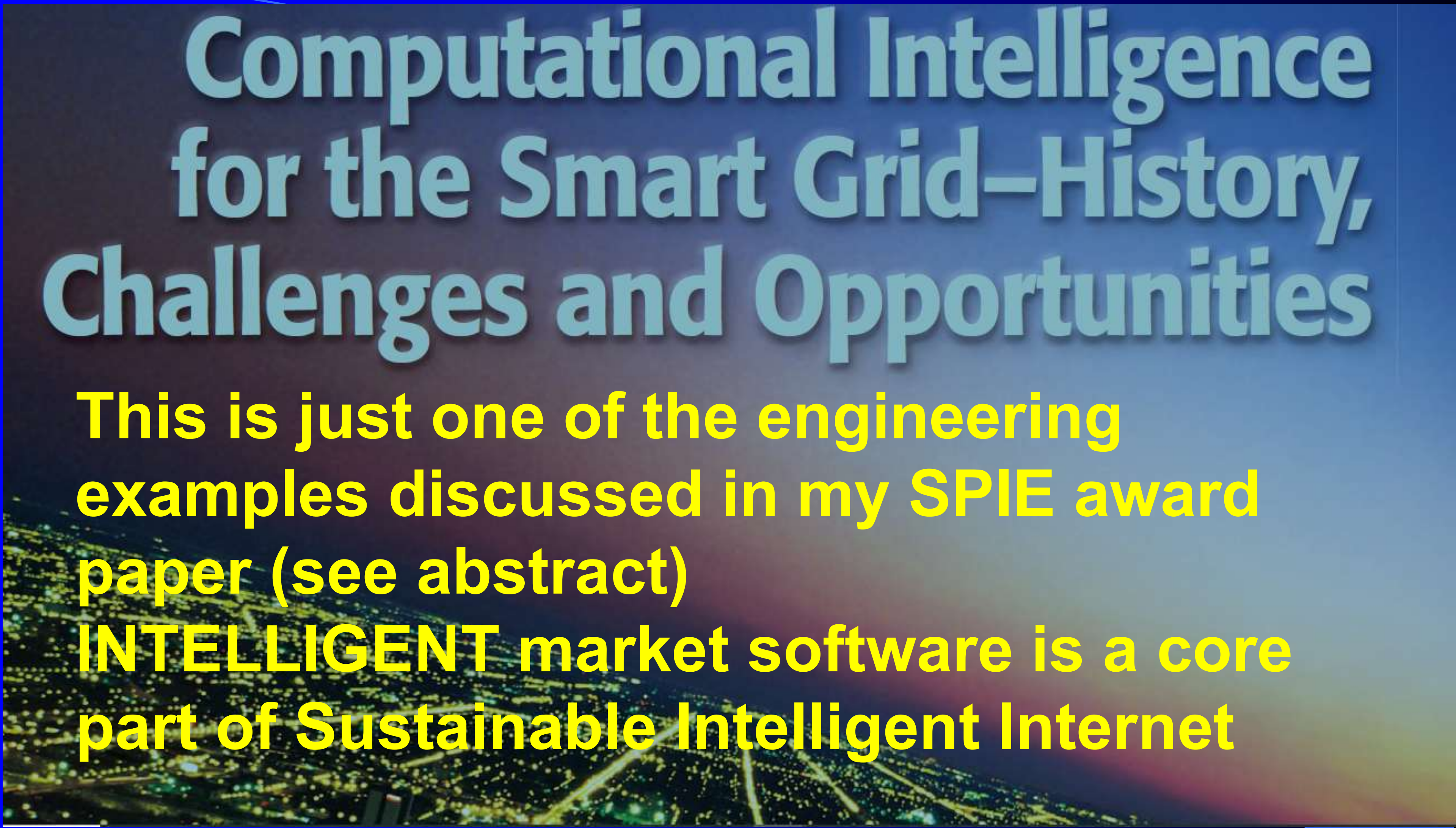

This is just one of the engineering examples discussed in my SPIE award paper (see abstract)

INTELLIGENT market software is a core part of Sustainable Intelligent Internet

IEEE COMPUTATIONAL INTELLIGENCE MAGAZINE
AUGUST 2011

Also posted at www.Werbos.com/energy.htm

The entire world economy WILL SOON BECOME just one of the apps on the Internet: a huge shift in human history

Most of
Earth History

DNA
Evolution:
Biology
(Including
Brains,
Families)
Rule Life

Learning Pains and
Unfinished Struggle
2000 BC to 2000AD

DNA,
Biology

??

New Global Controlling
Systems as Important
as DNA and $$

**Global Information
Technology Systems
Big Choices Being
Made Here and Now**

**Now Money is a SUBSYSTEM
Of INTERNET**

$$$$$$:
Struggle for
Effective
Market
Systems versus
Dystopic Problems

**?? Enable More,
Deeper, More Effective
Human Collective
Consciousness?**

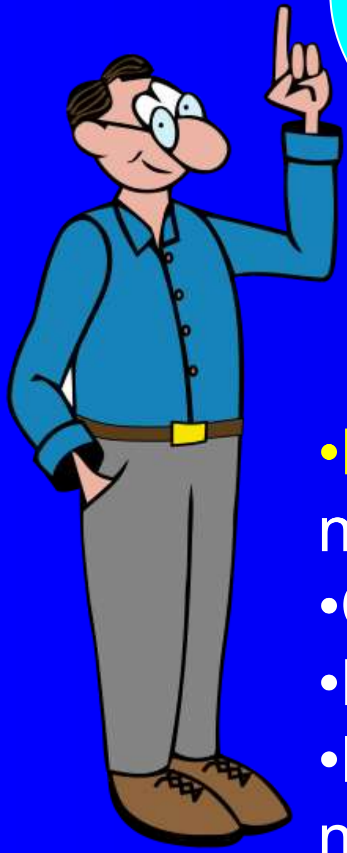

The new AI based on deep learning is remaking the world here and now. We need to jump to CNN and RNN, the next big thing.

- Deep learning = backprop + convolutional neural network (+bottleneck nets) + tricks
- CNN = cellular neural network (Chua/Roska)
- RNN: huge symposium in Barcelona December 2016
- New evidence that the brain is an “artificial neural network” (Frontiers in Systems Neuroscience)
- Huge choices here and now in where humanity goes next -- huge new risks, new technology options

Regular Cycles of Forward and Backward Signal Propagation in Prefrontal Cortex and in Consciousness

Paul Werbos and Joshua Davis

Frontiers in Systems Neuroscience

November 28, 2016

Open access, link posted at www.werbos.com/Mind.htm

From Brain to Mind: What Can We Learn Of Use Beyond the Level of the Mouse Brain?

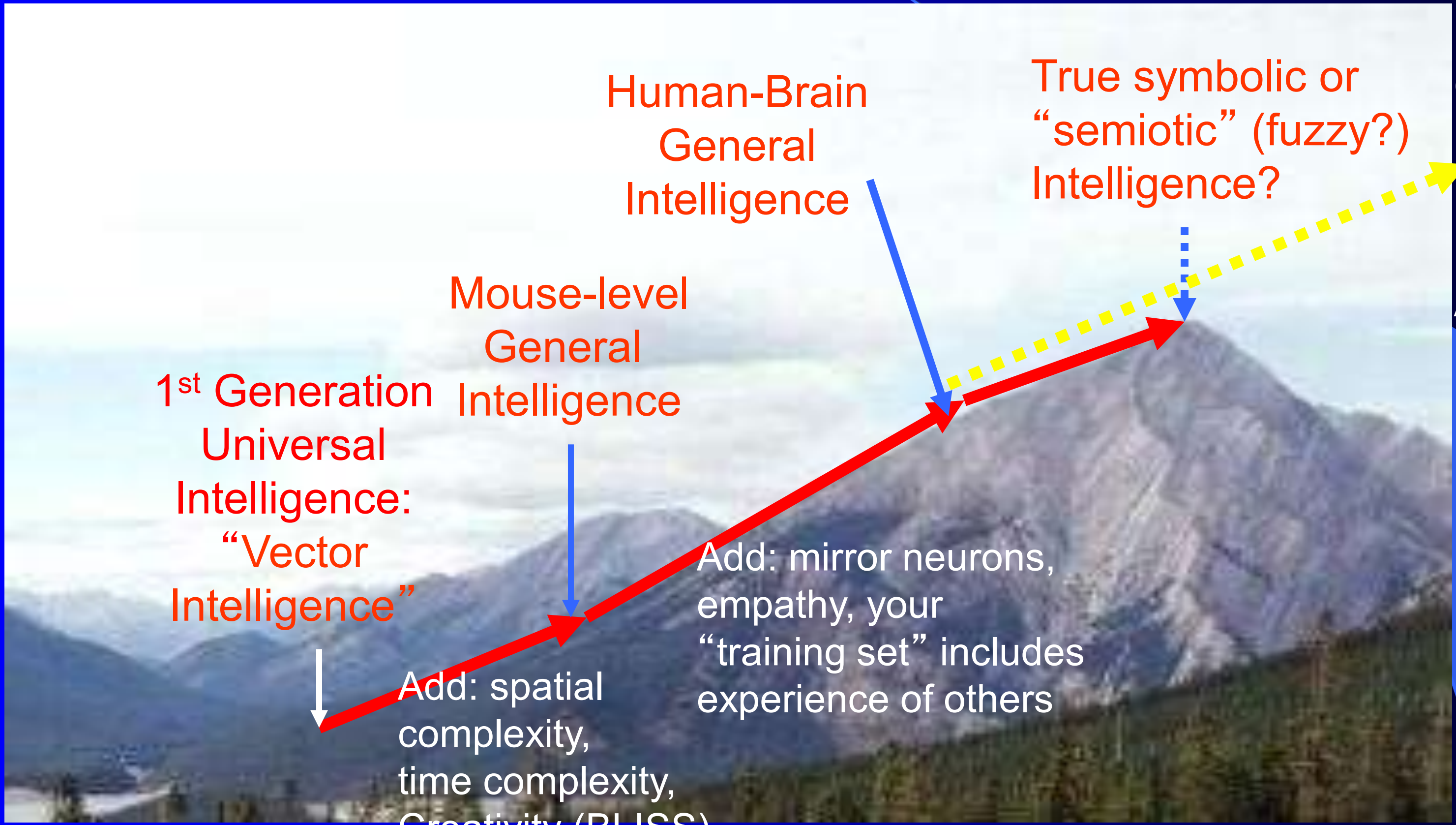

www.werbos.com/pi/Confucius_talk.pdf

And Neural Networks 2012; arxiv MLCI

Thermal Quantum Annealing (tQuA) for Quantum Artificial General Intelligence (QAGI) for Electricity

- Intelligent power grid requires dynamic optimization and adaptive forecasting networks, whose training is an optimization challenge
- tQuA offers many orders of magnitude improvement in such optimization tasks beyond Quantum Turing Machines (QTM) or older D-Wave
- Builds on earlier NSF Engineering work on adaptive & intelligent systems (AIS), power grid research, and quantum models for electronics QMHP

About 10 Independent Systems Operators (ISOs) Run the US Grid

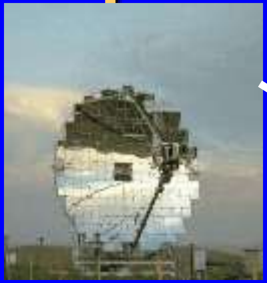

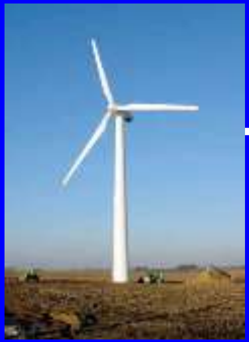

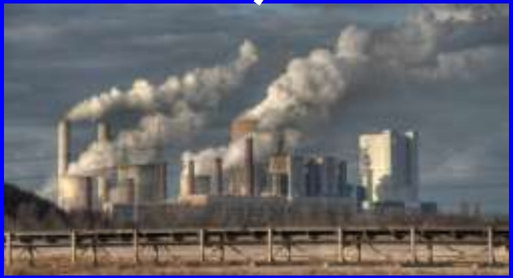

Transco
like IOU

ISOs Decide:

- Unit Commitment (Contracts to Generators a Day Ahead) & Advance Planning
- Economic Dispatch (Generators Used and Loads & Prices every 15 minutes)

Balancing Authorities:

Regulation Functions (every 2 seconds): stability, V, υ

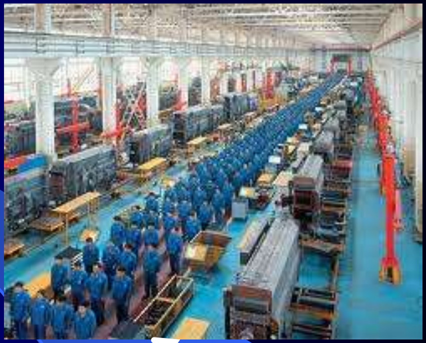

- Big Loads

Distribution Companies Discos

- ?

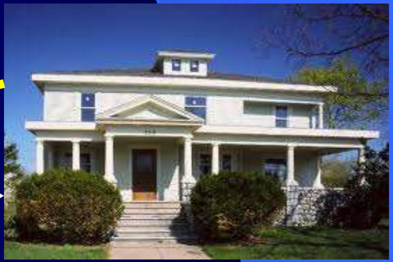

- Small Loads

- See www.ferc.gov, event calendar, June 2010

Time of day and predictability are crucial

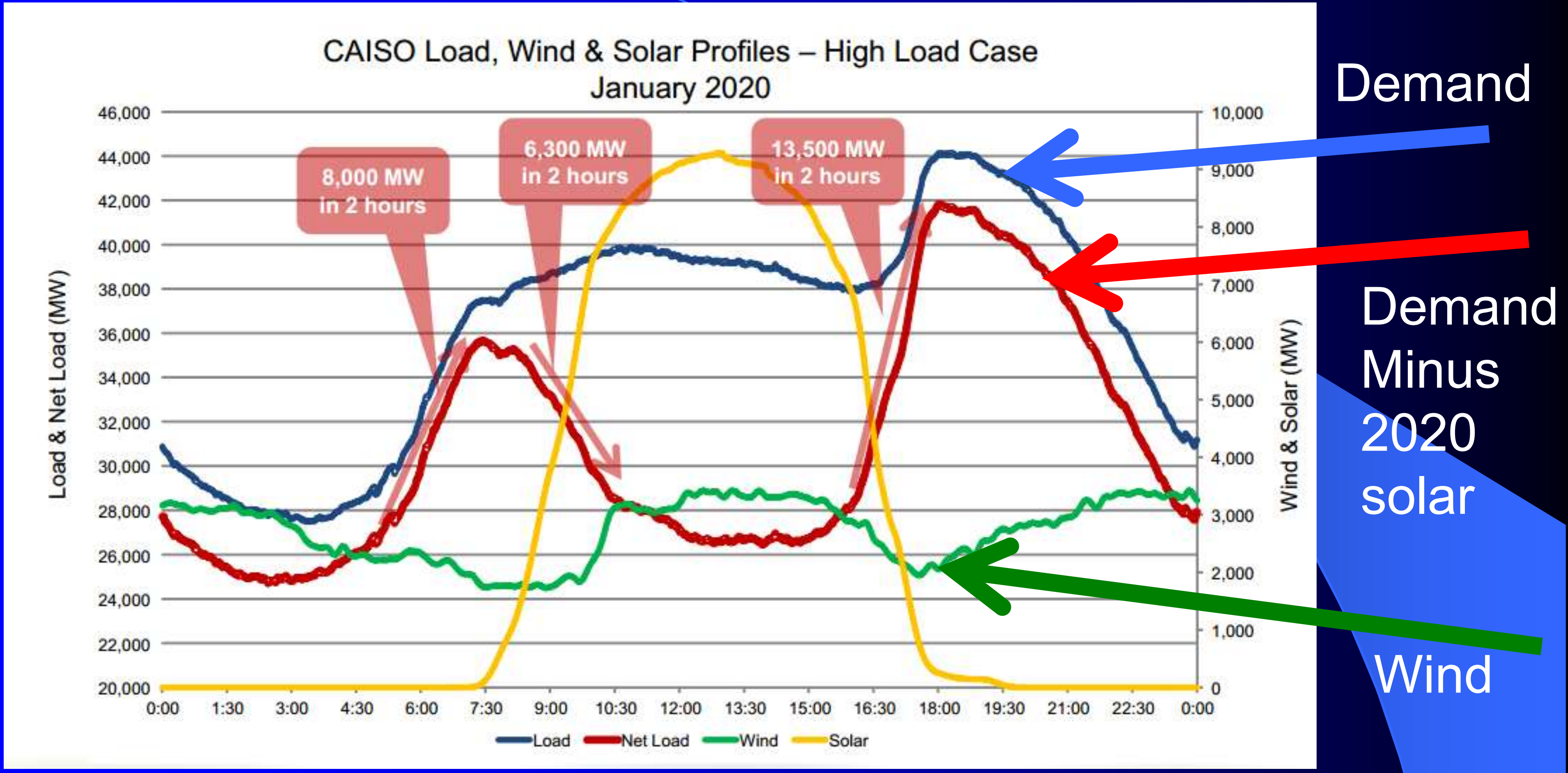

- Chile and Brazil have unique hydro base, so intelligent timing of its use avoids need for more storage
- Most of rest of world faces tricky choices, 10¢ extra

Biggest Opportunities for RLADP in US Power Grid and Many Others

- ISO Level: **Cut Cost of Renewables in Half**
  – FORESIGHT: From Optimal Power Flow to DSOPF, allows optimal management of storage, dams, time of day
  – Speed: Implementation on Neural Net Chips, allows optimization every second, not every 5 minutes. Control of power electronics turns wind from cost to asset
- Distribution Level: Enable Optimal Control
  – Use meshed networks, **handle electric cars and PHEV**
- User Level: (Mannheim) Intelligent Demand Response Can Accommodate Even Wind

Dynamic Stochastic Optimal Power Flow (DSOPF): How to Integrate the "Nervous System" of Electricity

- DSOPF02 started from EPRI question: can we optimally manage&plan the whole grid as one system, with foresight, etc.?
- Closest past precedent: Momoh's OPF integrates &optimizes many grid functions – but deterministic and without foresight. UPGRADE!
- ADP math required to add foresight and stochastics, critical to more complete integration.
- New work may deeply cut cost of hooking up solar (e.g. JTEC!) to electric power grids. Can we get do enough to get GE or ABB to follow through?

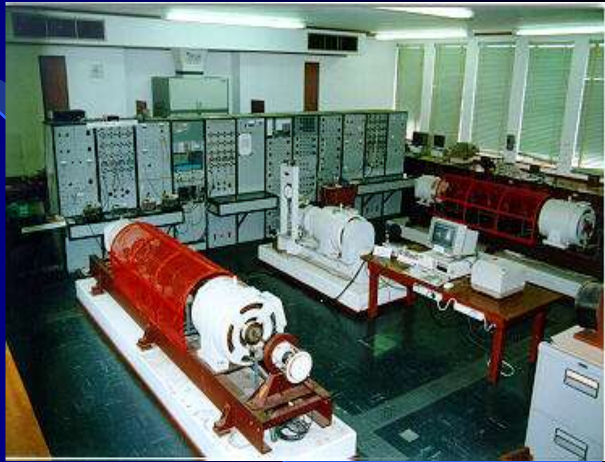

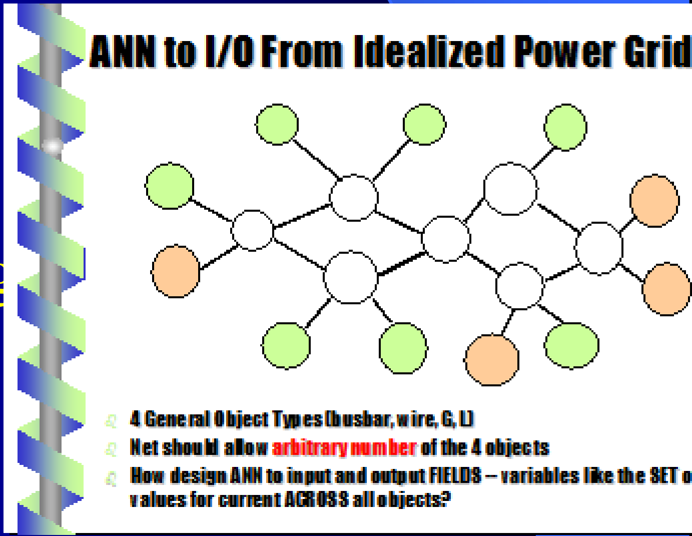

If we do DSOPF every two seconds, we can turn wind from liability to asset to grid

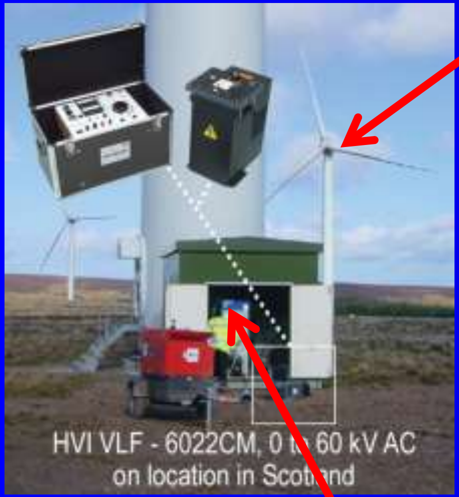

Today wind fluctuations cause lots of fluctuation second-to-second, raising cost for “regulations services,” “AGC.” Add 5 cents to a 10 cents real cost and you get 15 cents per kwh.

Wind farms have lots of power electronics. Optimal control of that electronics every two seconds can change the costly fluctuation into a big asset, a provider of service. Cut 3 cents from the net cost, and you get 7 cents per kwh net.

Liang, Harley and Venayagamoorthy (COPN): use massively parallel chips implementing neural ADP to get to this massive speedup.

New Breakthrough Opportunity: Liang, Harley, Venayagamoorthy

- Can solve AC-OPF and add foresight every 2 seconds instead of every 15 minutes using:
  - DSOPF for the foresight
  - New Neural Networks to handle large problems
  - Massive scaleable chips for speed, using neural networks
- When we do optimization at the same time scale as we stabilize the grid, we can get net benefit from wind and solar (telling their power converters what to do), changing cost to benefit

True quantum power (Q>=40) for true annealing requires low decoherence hardware: 4 threads in parallel

- Quantum optics. Can prototype design concepts with more accuracy and control, resolve important debates about how the systems really work? E.g Yeshua Davis's @Auckland Hoogerland/Carmichael
- Squids (superconductor loops, developed at NSA but more advanced in China. Dwave does not use in way that protects entanglement long enough)
- Quantum Dots, like Samsung TV. Needs new controls, but no known limit at Q<billion.
- Hi Speed digital design, high Q, leads beyond QuA

Fundamental Design for QuATh Quantum Annealing of Things

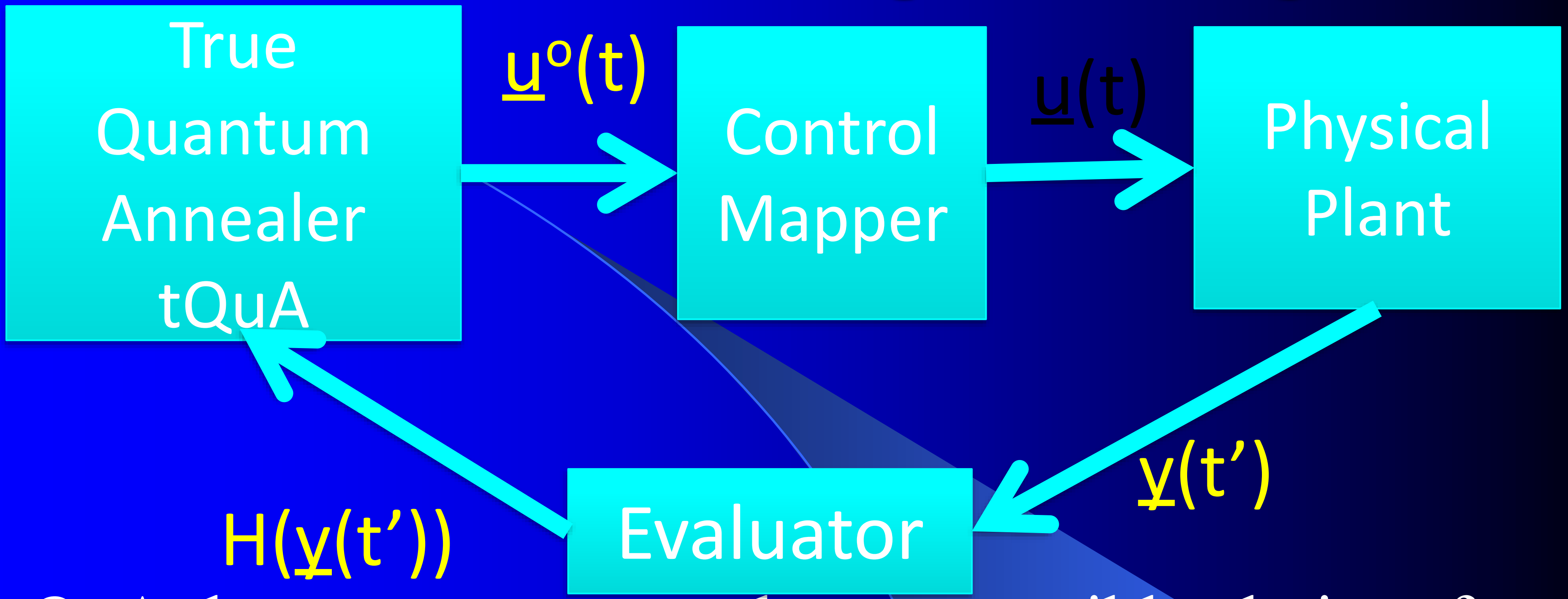

QuA does quantum search over possible choices for $\underline{u}(t)$, running from an initial time t_0 to a final time t', finds the one which maximizes H(t'). Just build the Mapper and Evaluator and connect the boxes!
